# Narrow prototypes of Asian subgroups in the United States: Implications for the Stop Asian Hate movement

**DOI:** 10.1177/13684302241305368

**Published:** 2025-01-16

**Authors:** Samantha R. Pejic, Jason C. Deska

**Affiliations:** Toronto Metropolitan University, Canada

**Keywords:** Asians, prototypes, social justice movements, stereotypes

## Abstract

The Stop Asian Hate movement is a collective for several anti-Asian-violence rallies and organizations in the United States (US). Research indicates that when asked to think about who is Asian, Americans’ prototype primarily comprises East Asian individuals (e.g., people from China, Japan, Korea) at the exclusion of people from other regions of Asia (e.g., South Asia). The current work extends this prototypicality research to examine implications for social justice movements. We focused on the Stop Asian Hate movement, which was designed to raise awareness and protest racial discrimination directed towards Asian Americans, particularly in light of COVID-19. Three studies tested whether people’s prototypes regarding who is Asian influenced who they believe is represented by the Stop Asian Hate movement, as well as potential implications of this bias. Compared to South Asians, people judged East Asians as more represented by the Stop Asian Hate movement (Study 1). When described as being the victim of a hate crime, participants perceived East Asian targets to be more credible, more traumatized, and their reporting of the crime on the SAAPI website was deemed more appropriate, compared to South Asian targets (Studies 2–3), effects that were mediated by judgments of prototypicality (Study 3).

Asian Americans comprise a significant proportion of the U.S. population (Budiman & Ruiz, 2021). However, despite the heterogeneity among Asian subgroups, they are monolithically categorized under the broad racial label of “Asian,” both colloquially and in census data (United States Census Bureau, 2024). This overgeneralization not only limits the informative value of census data, but also overlooks the diversity among Asian subgroups. Although only 40% of the Asian American population is East Asian (Budiman & Ruiz, 2021), individuals in the United States (US) believe that this Asian subgroup is more prototypically Asian compared to others, particularly compared to South Asians (Goh & McCue, 2021). Problematically, narrow prototypes have significant implications for whether victims are believed, as well as for perceptions of how psychologically harmed they are following victimization (Goh et al., 2022). Recognizing the recent spike in anti-Asian violence in the US during the COVID-19 pandemic (Stop Asian American Pacific Islander Hate [SAAPI], 2021), we aimed to understand if the narrow racial prototypes of Asian subgroups translate into beliefs about who is considered represented by social justice movements (i.e., the Stop Asian Hate movement), and implications of such bias. Specifically, we aimed to explore whether low-prototypical Asian Americans may not be believed to the same extent as high-prototypical Asian Americans when they experience and report identical acts of racism. Further, we tested how this bias may result in low-prototypical Asian Americans being excluded from social movements intended to support all Asian Americans (i.e., Stop Asian Hate movement).

Given previous research demonstrating the implications of narrow prototypes for beliefs about reporting victimization (Goh et al., 2022; for a review, see Kaiser et al., 2022), the current work examined whether cultural beliefs related to perceptions of who is considered “Asian” biased beliefs about targets’ credibility, level of harm experienced, and appropriateness of use of racially specific reporting outlets following hate-crime victimization. To that end, we first briefly outline population information pertinent to Asian Americans. We next review the relevant prototypicality literature, highlighting the conceptual overlaps between previously researched groups and the current population of focus. Finally, we discuss the Stop Asian Hate movement and its connection to the prototypicality literature.

## Asian Americans

The Asian population in the US is substantial and diverse. The most recent census data suggest there are over 22 million Asian Americans in the US who trace their roots to over 20 countries across Asia. Notably, Asians are the nation’s fastest growing racial or ethnic group, with the population rapidly growing by approximately 81% between 2000 and 2019 (Budiman & Ruiz, 2021). Despite the immense heterogeneity among Asians, they are often monolithically categorized, both colloquially and in census data (United States Census Bureau, 2024). Indeed, Asia is the largest and most populous continent in the world, comprised of 48 countries and three territories (WorldAtlas, 2021). The United Nations (UN) divides Asia into five subregions: East Asia, Southeast Asia, South Asia, Central Asia, and West Asia, all with drastically different populations and cultures. The broad Asian racial label not only overlooks the diversity among different Asian cultures, but also drastically reduces the informative value of census data. For example, although census data indicate there are over 22 million Asian Americans from over 20 countries, 85% of the Asian American population in the US are from six origin groups (Budiman & Ruiz, 2021). Specifically, Chinese Americans are the largest origin group (5.4 million), accounting for 24% of the Asian population in the US, followed by Indian Americans (4.6 million) and Filipinos (4.2 million), who account for 21% and 19% of the Asian population, respectively. Vietnamese (2.2 million), Korean (1.9 million), and Japanese Americans (1.5 million) respectively account for 10, 9, and 7% of the Asian population. Critically, despite the shared experience of being categorized under the same broad racial label, individuals from these backgrounds drastically differ in their appearance, language, and culture. One additional way in which they differ is the extent to which individuals in the US consider each of them Asian (Goh & McCue, 2021).

## On Narrow Prototypes

Past research demonstrates that, in the US, people deem East Asians more prototypically Asian and less foreign compared to other Asian subgroups (Goh & McCue, 2021). Notably, despite having similar population levels in the US (Budiman & Ruiz, 2021), South Asians are deemed significantly less prototypically Asian, and significantly more foreign than East Asians. Researchers explain this finding through the phenomenon described as the disjuncture between ingroup and outgroup racial assignment (J. Lee & Ramakrishnan, 2020). Simply, there is a gap between bureaucratic considerations of the Asian category and lay Americans’ understanding of it. Although the U.S. Census Bureau defines the Asian racial category as inclusive of all five subregions of Asia, for most Americans, East Asians are who is considered Asian (J. Lee & Ramakrishnan, 2020). This narrow racial assignment of East Asian as the default for Asian has been demonstrated among White, Black, Latino, and most Asian Americans, with the notable exception of South Asians (who do not perceive East Asians as the default), deemed the South Asian exclusion.

However, disjuncture in racial assignment is not unique to the US. Contrary to the US, in the United Kingdom (UK), the prototypical racial assignment for Asian is South Asian (Goh & McCue, 2021). Because prototypes are culturally determined, it follows that who is considered prototypical in one culture may not be so in another culture (Turner et al., 1987). One explanation provided for the difference in the default Asian racial assignment between the US and the UK is related to different historical immigration patterns and current population estimates (Goh & McCue, 2021). In the US, East Asians comprised the first mass immigration from Asia and now make up the largest Asian subgroup (Goh & McCue, 2021; E. Lee, 2015; Budiman & Ruiz, 2021). In contrast, South Asians comprise the largest Asian subgroup in the UK (Office for National Statistics, 2024), and were, historically, the dominant Asian subgroup immigrating into the UK (Visram, 2002).

This default racial assignment of East Asians in the US, which we refer to as the Asian prototypicality bias, demonstrates a very narrow prototype regarding who is considered Asian. Prototypes encompass culturally and contextually determined attributes, such as physical traits, behaviours, and attitudes (Rosch, 1978) that serve as heuristics for perceivers (Devos & Banaji, 2005; Devos & Mohamed, 2014; Goh & McCue, 2021; Kunstman et al., 2016). They allow individuals to simplify groups into overarching mental representations (Brewer, 1988; Rosch, 1978; Turner et al., 1987). Although prototypes may be cognitively useful, holding particularly narrow prototypes carries serious implications for those deemed not prototypical, such that individuals who do not match the prototype of their social group are often disliked, forgotten, or punished (Phelan & Rudman, 2010; Purdie-Vaughns & Eibach, 2008; Schug et al., 2017; Sesko & Biernat, 2010; Vogel et al., 2021). An intersectional approach to prototypes (Crenshaw, 1989) provides another avenue for understanding how prototypes can be detrimental. This perspective outlines how individuals who possess devalued intersectional identities are deemed nonprototypical of their broader social categories and experience intersectional invisibility (Kaiser et al., 2022; Purdie-Vaughns & Eibach, 2008). For example, Black women, lesbian women, and older women are often not fully recognized as belonging to their gender group due to possessing nonprototypical characteristics related to age, sexuality, and race. Intersectional invisibility results in the dismissal of contributions by individuals with intersectional identities, as well as their claims of bias (Purdie-Vaughns & Eibach, 2008).

Narrow prototypes also hold significant implications for individuals who are victimized. For example, when described as claiming to be the victim of a sexual assault, women deemed as less gender-prototypical by participants were less likely to have their claims believed, and the harassment itself was deemed as less psychologically harmful compared to that suffered by more prototypical looking women (Goh et al., 2022). Further, bystander intervention is a critical component to sexual harassment through lessening the burden for targets (Dobbin & Kalev, 2019; Fitzgerald & Cortina, 2018). However, compared to White women, when Black women were presented as at risk of sexual assault, participants reported less intent to intervene (Katz et al., 2017). The consequences of prototypicality biases in sexual assault victimization are not limited to laypersons—jurors and police are also more likely to discount the seriousness of sexual assault against Black women compared to White women (Coker et al., 2015; Foley et al., 1995). Further, Black sexual assault victims are more likely to receive substandard care, and to experience discrimination and revictimization from medical professionals and police officers compared to White victims (Coker et al., 2015; Jacques-Tiura et al., 2010). Together, similar effects on credibility and victim treatment may be present for nonprototypical Asians who are the victims of race-based hate crimes.

## Anti-Asian Discrimination and the Stop Asian Hate Movement

Understanding if the implications of narrow prototypes for victims’ credibility extend to other Asian subgroups is now more important than ever. The COVID-19 outbreak resulted in substantial increases in incidences of racism, discrimination, and violence against Asian Americans (Croucher et al., 2020), particularly Asian American elders (New York City Commission on Human Rights, 2022). As COVID-19 cases and deaths increased in the US, so too did prejudice against Asian Americans. Explanations for why Asian Americans became the targets of increased discrimination, harassment, racial slurs, and physical attacks because of the pandemic are often rooted in the belief that the virus originated in China—a belief perpetuated not only by the media, but also by government officials (Croucher et al., 2020).

This spike in Anti-Asian hate crimes during the COVID-19 pandemic galvanized the Stop Asian Hate movement. Although there is no singular official definition, this movement is often described as the umbrella name of several rallies and demonstrations against anti-Asian violence that have been held across the US since 2021 in response to racism and the rise of violence and hate crimes against Asian Americans, specifically related to the COVID-19 pandemic. There are also additional initiatives associated with the movement, such as SAAPI, which tracks violence and harassment against^1^Asian Americans and Pacific Islanders in the US (SAAPI, 2021). In addition to building networks of culturally competent organizations across Asian communities, providing resources to local leaders, and working with partners to change how issues of racism are understood and discussed, the SAAPI coalition provides an online reporting system, available in six different languages, dedicated to anti-AAPI hate incidents.

Although governmental blame for the spread of COVID-19 was uniquely placed on China, other Asian subgroups, such as those with families from Korea, Vietnam, and the Philippines, among others, also faced growing racism because of the pandemic (Tavernise & Oppel, 2020). The overall increase in anti-Asian violence against other non-East Asian subgroups further reinforces the Asian prototypicality beliefs in the US, such that, in the eyes of many Americans, Asian is equivalent to East Asian. The rise of anti-Asian hate crimes because of the pandemic demonstrates the prevalence of bigotry, xenophobia, and racism in the US. In addition, the occurrence of indiscriminate anti-Asian violence towards all Asian subgroups, unfortunately, demonstrates the real-life consequences of prototypes.

Racial assignment in the US includes both racial self-identification (how an individual identifies themselves) and observed race (how individuals are identified by another; J. Lee & Ramakrishnan, 2020). However, self-identification and observed race do not always correspond, resulting in racial mismatch (Massey, 2009; Mora, 2014; Roth, 2018). Importantly, the consequences of racial mismatch are conceptually related to those of prototypicality, such that when an individual racially self-identifies in a way incongruent with how they are observed, they face similar instances of discreditation and hostility as do individuals who are not prototypical of the social category they identify with (Goh et al., 2022; J. Lee & Ramakrishnan, 2020).

One of the major components of the SAAPI coalition is the online reporting feature, but it is unclear how non-East Asians using the feature are received. A similar question applies to the Stop Asian Hate movement broadly. Due to the rise in anti-Asian violence during the pandemic, many individual cities across the US implemented websites and sectors of police dedicated to anti-Asian hate crimes (e.g., New York City, San Francisco). However, given the narrow prototypes of Asians demonstrated in the US, there is the potential that non-East Asian individuals using such resources will experience negative encounters if deemed nonprototypically Asian. As many non-East Asians have been the victim of anti-Asian hate crimes, this poses both a significant probability and a concern (Tavernise & Oppel, 2020). Understanding whether the Asian prototypicality bias in the US extends to who is deemed represented by the Stop Asian Hate movement, and the implications of this bias for victimization reporting, is crucial in highlighting the serious, real-word consequences of narrow prototypes and will allow us to begin to understand how to best dismantle them.

## Current Work

The Stop Asian Hate movement aims to support people regardless of their prototypicality. However, due to the influence of prototypicality biases on victim reporting metrics (Goh & McCue, 2021), it is possible that low-prototypical Asian Americans (i.e., South Asians) may not be believed when experiencing and reporting hate crimes to the same extent as high-prototypical Asian Americans (i.e., East Asians). In addition to the psychological toll incurred when denied credibility, it is further possible that this bias may translate into low-prototypical Asian Americans being denied membership in social justice movements compared to their high-prototypical counterparts.

As such, we tested whether participants would make biased judgments about which targets are represented by the Stop Asian Hate movement; whether credibility, appropriateness, and trauma ratings following hate crime victimization would be influenced by target race; and whether beliefs about Asian prototypicality potentially underlie these racial biases. Given that East Asians and South Asians comprise the largest Asian subgroups in the US (Budiman & Ruiz, 2021), and previous research has compared Asian prototypicality ratings between these two Asian subgroups (Goh & McCue, 2021), we focused on these two groups in the current studies. We hypothesized that participants would judge East Asians as more represented by the Stop Asian Hate movement than their South Asian counterparts. We predicted a similar pattern for credibility, appropriateness, and trauma ratings. Specifically, following claims of being the victim of a hate crime, compared to South Asian targets, participants would be more likely to believe East Asians were the victim of a hate crime, feel it is more appropriate for them to report the incident on the SAAPI online reporting system, and indicate they would be more traumatized if they were the victim of a hate crime. Finally, we examined whether beliefs about representativeness by the Stop Asian Hate movement would underlie these racial biases. We hypothesized that participants would deem East Asians as more credible, traumatized, and more appropriate for them to use the SAAPI reporting system to the extent they are judged as more represented by the Stop Asian Hate movement.

We tested these hypotheses across three studies. In each, participants were provided a definition of the Stop Asian Hate movement and then viewed images of targets that differed in race and gender. In Study 1, participants assessed the extent to which they would classify each target as represented by the movement. In Study 2, participants viewed target images and read a brief vignette describing the target as claiming to be the victim of a hate crime, and that they were intending to report the crime on the SAAPI online reporting system. Participants reported how much they believed the target was the victim of a hate crime, how appropriate they believed it was that the target used the SAAPI website specifically, and how traumatized they believed the target would be if they were the victim of a hate crime. Finally, Study 3 tested whether differential beliefs about representativeness by the Stop Asian Hate movement mediated perceptions of targets’ credibility, appropriateness, and level of traumatization after being the victim of hate crime.

## Study 1

Previous research suggests that, in the US, East Asians are more prototypically Asian than South Asians. Based on these findings, the goal of Study 1 was to test if this demonstrated Asian prototypicality bias conceptually extended to whom participants deemed as most represented by the Stop Asian Hate movement. To do this, we asked participants to indicate the extent to which they would classify East Asian, South Asian, and White men and women as represented by the Stop Asian Hate movement. We showed participants 30 images of targets that differed in race (i.e., East Asian, South Asian, White) and gender (i.e., man, woman), in a randomized order, and asked them to assess the extent to which they would classify the target as represented by the Stop Asian Hate movement, using procedures similar to those outlined in previous research (Goh & McCue, 2021). Of primary interest was the comparison of representativeness ratings between East Asian and South Asian targets. Specifically, we hypothesized that participants would perceive East Asians as more represented by the Stop Asian Hate movement compared to South Asians. We also included White targets as a reference group. Although targets comprised both men and women, our a priori theorizing, hypotheses, and analytic plan focused on race. Readers interested in gender effects can find them on the Open Science Framework (OSF; https://osf.io/nmr25/?view_only=9e5a33858c624c5089dd4a9bc7944aa0).

### Method

#### Statistical power and participants

We determined the sample size for Study 1 based on the sample size used in a conceptually similar study in the literature (Goh & McCue, 2021). Using this reference point, we targeted at least 150 participants. For all studies, participants were recruited via CloudResearch (Litman et al., 2017), which is an opt-in, web-based survey platform known to have good data quality (Douglas et al., 2023). All participants over 18 years old who lived in the US were eligible to participate, and no participants were excluded from analyses. Data for all studies were collected prior to data analysis, and all measures, manipulations, and exclusions are reported in these studies (https://osf.io/nmr25/?view_only=9e5a33858c624c5089dd4a9bc7944aa0). A sensitivity analysis revealed that when examining the difference between two dependent means, Study 1 provided 80% power to detect an effect size of *d* = 0.23.

A total of 151 CloudResearch workers (*M*_age_ = 37.59, *SD*_age_ = 10.76; 59.6% women, 39.1% men, 1.3% nonbinary; 70.2% White, 11.9% Black/African American, 6.0% bi-racial, 4.6% East Asian, 1.3% Southeast Asian, 0.7% West Asian) participated in the study.

#### Materials

Stimuli comprised photographs of 10 East Asian, 10 South Asian, and 10 White targets sourced from the Toronto Ethnically Diverse Face Database (Latif et al., 2024). Within each racial group, five photos were of women and five were of men. All targets had neutral expressions, were in colour, and were configured at 600 width × 400 height.

#### Procedure

After providing informed consent, participants were provided the following definition of the Stop Asian Hate movement:

This movement is described as the umbrella name of several rallies and demonstrations against anti-Asian-violence which have been held across the United States in 2021 in response to racism and the rise of violence and hate crimes against Asian Americans related to the COVID-19 pandemic. There are also specific initiatives associated with the movement, such as the Stop Asian American Pacific Islander Hate (SAAPI) that tracks violence and harassment against Asian-Americans and Pacific Islanders in the United States. (SAAPI, 2021)

After reading the definition, participants saw all 30 photos in a randomized order. For each photograph, participants were asked to indicate how likely they would be to classify the target as represented by the Stop Asian Hate movement, on a 7-point Likert scale (1 = *not at all likely*, 7 = *extremely likely*). After completing the prototypicality rating, participants continued to the next trial. Following the completion of all 30 ratings, participants were asked to indicate how familiar they were with the Stop Asian Hate movement on a 7-point Likert scale (1 = *not familiar at all*, 7 = *extremely familiar*). Finally, participants completed a demographic survey (e.g., age, racial identity, gender) and were fully debriefed.

### Results

**Figure 1.**
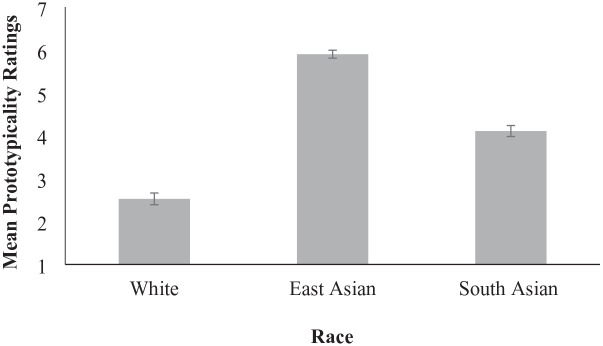
Perceived representativeness by the Stop Asian Hate movement by Asian subgroup: Study 1. *Note.* Error bars indicate standard error of the mean.

We first averaged participants’ prototypicality ratings separately by race to create composite scores for each group. Because our primary interest was the extent to which participants believed the East Asian targets were more represented by the Stop Asian Hate movement compared to South Asian targets, we analysed results using paired-samples *t* tests (see Figure 1). Consistent with hypotheses, participants viewed East Asians (*M* = 5.89, *SD* = 1.14) as more represented by the Stop Asian Hate movement compared to South Asian targets (*M* = 4.11, *SD* = 1.58), *t*(150) = 12.18, *p* < .001, *d* = 0.99, 95% CI [1.50, 2.08].^2^ Unsurprisingly, participants judged East Asian targets as more represented than White targets (*M* = 2.53, *SD* = 1.68), *t*(150) = 17.74, *p* < .001, *d* = 1.44, 95% CI [2.99, 3.75], and South Asian targets as more represented than White targets, *t*(150) = 10.50, *p* < .001, *d* = 0.85, 95% CI [1.28, 1.88]. Participants reported relatively high familiarity with the Stop Asian Hate movement (*M* = 4.39, *SD* = 1.71). Reliability analyses for the 10 images included in each condition support excellent internal consistency for the East Asian (α = .95), South Asian (α = .97), and White conditions (α = .98).

### Discussion

Study 1 provides initial evidence consistent with our hypothesis. Compared to South Asian targets, East Asian targets were perceived to be more represented by the Stop Asian Hate movement. Perhaps unsurprisingly, participants viewed both groups as more represented by the Stop Asian Hate movement than White targets. Although seemingly obvious, this is not necessarily trivial. Rather, it shows that although participants viewed South Asian targets as less representative of the Stop Asian Hate movement than East Asian targets, they still saw them as more represented than a group that clearly is not.

## Study 2

In Study 1, participants indicated that East Asians are more represented by the Stop Asian Hate movement compared to South Asians. In Study 2, we investigated several implications of these beliefs. Specifically, we tested the extent to which judgments of credibility, harm, and appropriateness of reporting on the SAAPI website following claims of hate crime victimization were influenced by target race.

We measured both credibility and psychological harm because recent work demonstrates the influence of narrow prototypes on both variables. For instance, individuals deemed less prototypically “woman” are perceived as less credible, and less psychologically harmed following sexual assault victimization, compared to more visually prototypical women (Goh et al., 2022). As narrow prototypes regarding which Asian subgroups are more prototypically “Asian” have been demonstrated among U.S. participants, there is a clear reason to predict both credibility and harm would be influenced by target race. Further, as Study 1 conceptually replicated past findings of the prototypical perception of East Asians as “Asian,” and further extended this bias to whom participants believed to be most represented by the Stop Asian Hate movement, there is also a clear reason to predict this bias would influence whom participants believe should utilize websites dedicated to the movement. Therefore, we measured appropriateness of reporting a hate crime on the SAAPI website.

In Study 2, participants viewed six images of targets that differed in race (i.e., East Asian, South Asian, White) and gender (i.e., man, woman), and read a brief written vignette depicting the target as claiming to be the victim of a hate crime and further planning to report the incident on the SAAPI website. Using procedures similar to those outlined in previous research (Goh et al., 2022), we then asked participants to indicate the extent to which they believed the target was the victim of a hate crime, how traumatized they thought they would be, and how appropriate they believed it was that the target was choosing to report the crime on the SAAPI website. We hypothesized that participants would perceive East Asians as more credible, more traumatized, and that it was more appropriate for them to use the SAAPI website compared to South Asians targets. Comparisons to White targets are included as a reference but were not of primary interest.

### Method

#### Statistical power and participants

We determined the sample size for Study 2 based on the sample size used in a conceptually similar study in the literature (Goh & McCue, 2021). Using this reference point, we targeted approximately 150 participants. All participants over 18 years old who lived in the US were eligible to participate, and no participants were excluded from analyses.

A total of 149 CloudResearch workers (*M*_age_ = 37.79, *SD*_age_ = 11.04; 32.88% women, 66.44% men, 0.67% nonbinary; 70.47% White, 7.38% Black/African American, 4.7% bi-racial, 0.67% East Asian, 0.67% Southeast Asian, 7.38% Latino) participated in the study. A sensitivity analysis revealed that when examining the difference between two dependent means, Study 2 provided 80% power to detect an effect size of *d* = 0.23.

#### Materials

Because we added several measures and a vignette, we showed participants fewer targets to reduce fatigue. For each Race × Gender combination, we selected targets with the highest average prototypicality score from Study 1 (see Table 1). Consequently, stimuli for Study 2 comprised six photographs (two East Asians, two South Asians, two White targets; one woman, one man), representing the highest prototypicality score for each condition from the original 30 images in Study 1.

**Table 1.** Prototypicality means by condition and image: Study 1.

Image number	Condition					
	East Asian women	East Asian men	South Asian women	South Asian men	White women	White men
	*M* (*SD*)	*M* (*SD*)	*M* (*SD*)	*M* (*SD*)	*M* (*SD*)	*M* (*SD*)
1	6.05 (1.33)	6.01 (1.28)	4.22 (1.71)	3.86 (1.86)	2.62 (1.92)	2.59 (1.81)
2	5.88 (1.38)	5.96 (1.41)	4.33 (1.81)	3.97 (1.77)	2.52 (1.92)	2.44 (1.82)
3	6.07 (1.28)	5.95 (1.41)	4.17 (1.84)	4.04 (1.68)	2.50 (1.93)	2.77 (1.75)
4	5.61 (1.35)	5.94 (1.43)	4.32 (1.87)	4.05 (1.80)	2.54 (0.91)	2.50 (1.81)
5	6.03 (1.24)	5.50 (1.52)	4.28 (1.79)	3.85 (1.79)	2.47 (1.87)	2.34 (1.80)

#### Procedure

We provided participants with the same definition of the Stop Asian Hate movement provided in Study 1. After reading the definition, participants saw all six photos in an independently randomized order. For each photograph, participants read a brief written vignette describing that the depicted target claimed they were the victim of a hate crime and was planning to report the incident on the SAAPI online reporting system. The following definition of a hate crime was provided: “A crime motivated by bias against race, colour, religion, national origin, sexual orientation, gender, gender identity, or disability” (United States Department of Justice, 2022). After viewing the image of the target as well as the written vignette, participants indicated how much they believed the target was the victim of a hate crime (credibility; 1 = *do not believe at all*, 7 = *definitely believe*), how traumatized they believed the individual would be if they were a victim of a hate crime (harm; 1 = *not traumatized at all*, 7 = *extremely traumatized*), and how appropriate they believed it was that the individual was reporting the incident on the SAAPI online reporting system (appropriateness; 1 = *not at all appropriate*, 7 = *extremely appropriate*). After completing all ratings, participants continued to the next trial. Following the completion of all trials, participants were asked to indicate how familiar they were with the Stop Asian Hate movement using the same measure as in Study 1. Finally, participants completed a demographic survey (e.g., age, racial identity, gender) and were fully debriefed.

### Results

We first averaged participants’ credibility, harm, and appropriateness ratings separately by race to create composite scores for each group. Of interest was the extent to which, when depicted as claiming to be the victim of a hate crime, East Asians’ claims were viewed as more credible, they were viewed to experience more harm as a result of the victimization, and it was viewed to be more appropriate for them to report their victimization on the SAAPI website, compared to South Asians. Because our primary concern was the comparison between East and South Asian targets, we again used paired-samples *t* tests to test the hypotheses.

As predicted, participants were more likely to believe East Asians were the victim of a hate crime (*M* = 5.78, *SD* = 1.29), compared to South Asians targets (*M* = 5.44, *SD* = 1.33), *t*(148) = 4.13, *p* < .001, *d* = 0.34, 95% CI [0.18, 0.50]. Further, when told the target was planning on reporting the crime to the SAAPI website, participants deemed it to be more appropriate for East Asians (*M* = 6.17, *SD* = 1.18) compared to South Asian targets (*M* = 5.37, *SD* = 1.69), *t*(148) = 6.83, *p* < .001, *d* = 0.56, 95% CI [0.56, 1.01]. However, when described as being the victim of a hate crime, no significant differences between participants’ perceptions of harm emerged between East Asian (*M =* 5.63, *SD* = 1.14) and South Asian targets (*M* = 5.56, *SD* = 0.86), *t*(148) = 0.97, *p* = .335, *d* = 0.08, 95% CI [−0.08, 0.22].

Unsurprisingly, White targets were perceived to be less credible (*M* = 3.38, *SD* = 1.70), compared to East Asian, *t*(148) = −14.84, *p* < .001, *d* = −1.22, 95% CI [−2.08, −2.71], and South Asian targets, *t*(148) = −13.73, *p* < .001, *d* = −1.13, 95% CI [−1.76, −2.35], respectively. A similar pattern emerged for appropriateness beliefs, such that it was deemed less appropriate for White targets (*M* = 3.11, *SD* = 1.82) to use the reporting system compared to East Asian, *t*(148) = −16.91, *p* < .001, *d* = −1.38, 95% CI [−2.70, −3.42], and South Asian targets, *t*(148) = −12.91, *p* < .001, *d* = −1.06, 95% CI [−1.92, −2.62], respectively. Participants perceived White targets (*M* = 4.23, *SD* = 1.77) to be significantly less traumatized if victimized by a hate crime compared to East Asian, *t*(148) = −10.03, *p* < .001, *d* = −0.82, 95% CI [−1.12, −1.67], and South Asian targets, *t*(148) = −10.01, *p* < .001, *d* = −0.82, 95% CI [−1.06, −1.59], respectively. Participants reported moderate familiarity with the movement (*M* = 3.89, *SD* = 1.95).

### Discussion

The results of the current study serve to conceptually replicate past findings supporting the implications related to narrow beliefs about prototypicality. East Asians are typically viewed as more prototypically “Asian” in the US than South Asians (Goh & McCue, 2021). When depicted as claiming to be the victim of a hate crime, East Asians’ claims were viewed as more credible, and it was viewed to be more appropriate for them to report their victimization on the SAAPI website, compared to South Asians. However, no significant differences in how traumatized the target would be emerged between South Asian and East Asian targets. Given this unexpected finding, we sought to test it again in Study 3.

## Study 3

Study 2 provided preliminary support for potential downstream implications of the observed prototypicality bias. Specifically, when depicted as being the victim of a hate crime, East Asians’ claims were viewed as more credible, and it was deemed more appropriate for them to use the SAAPI online reporting system compared to South Asians. Unexpectedly, no differences in beliefs about harm were observed between East and South Asian targets. Importantly, the hypotheses made regarding these differences were informed by the representativeness of the Stop Asian Hate movement effects observed in Study 1. Although beliefs that East Asians are more represented by the Stop Asian Hate movement theoretically support the results of Study 2, this was not empirically tested. Therefore, the primary goal of Study 3 was to test this prediction. Of interest was if perceptions of representation by the Stop Asian Hate movement predict perceptions of credibility, harm, and appropriateness of reporting on the SAAPI website following claims of hate crime victimization. A secondary goal of the current study was to replicate the findings of Studies 1 and 2.

Identical to Study 2, participants viewed six images of targets that differed in race (i.e., East Asian, South Asian, White) and gender (i.e., man, woman). We then asked participants to indicate the extent to which they would classify each target as represented by the Stop Asian Hate movement, using identical procedures as in Study 1. Following prototypicality ratings, participants read a brief written vignette depicting that the target was claiming to be the victim of a hate crime and was further planning to report the incident on the SAAPI website. Identical to Study 2, participants then indicated the extent to which they believed the target was the victim of a hate crime, how traumatized they thought they would be, and how appropriate they believed it was that the target was choosing to report the crime on the SAAPI website, specifically. We hypothesized that participants would perceive East Asians as more credible, more traumatized, and that it was more appropriate for them to use the SAAPI website compared to South Asians and White targets. It was further anticipated that perceptions of how represented the target was by the Stop Asian Hate movement would drive the relationship between race and all three dependent variables.

### Method

#### Statistical power and participants

To maintain consistency across studies, we targeted at least 150 participants for Study 3. All participants over 18 years old who lived in the US were invited to participate, and no participants were excluded from analyses.

A total of 150 CloudResearch workers (*M*_age_ = 39.51, *SD*_age_ = 11.28; 42% women, 56.67% men, 1.3% preferred not to specify; 78% White, 10% Black/African American, 2% bi-racial, 0.66% Indigenous, 1.3% South Asian, 1.3% East Asian, 5.3% Latino, 1.3% preferred not to specify) participated in the study. A sensitivity analysis revealed that when examining the difference between two dependent means, Study 1 provided 80% power to detect an effect size of *d* = 0.23.

#### Procedure

The procedure for Study 3 was identical to that for Study 2, with the notable exception of the additional representativeness measure used in Study 1 presented first for all targets.

### Results

The first goal of Study 3 was to replicate our findings from Study 1 by comparing perceptions of representativeness of the Stop Asian Hate movement between East Asian and South Asian targets. Identical to Study 1, we first averaged participants’ prototypicality ratings separately by race to create composite scores for each group. Of interest was if participants again believed that East Asian targets were more represented by the Stop Asian Hate movement compared to South Asian targets. Results of paired-samples *t* tests replicated those in Study 1, providing further support for the primary hypothesis. Specifically, participants viewed East Asian targets (*M* = 6.35, *SD* = 0.89) as more represented by the Stop Asian Hate movement compared to South Asian targets (*M* = 4.95, *SD* = 1.70), *t*(149) = 9.57, *p* < .001, *d* = 0.78, 95% CI [1.11, 1.68]. And, participants viewed White targets (*M* = 2.19, *SD* = 1.60) as less represented by the movement compared to East Asian, *t*(149) = 25.12, *p* < .001, *d* = 2.05, 95% CI [3.83, 4.48], and South Asian targets, *t*(149) = 15.84, *p* < .001, *d* = 1.29, 95% CI [2.42, 3.10], respectively.

The second goal of Study 3 was to replicate the implications of beliefs about East Asians’ prototypicality of the Stop Asian Hate movement by comparing perceptions of credibility, appropriateness, and trauma when depicted as being the victim of a hate crime between East Asian and South Asian targets. Identical to Study 2, to address this question, we first averaged participants’ credibility, harm, and appropriateness ratings separately by race to create composite scores for each group.

To first address credibility, results of paired-samples *t* tests replicated those found in Study 2. Specifically, participants were more likely to believe East Asians were the victim of a hate crime (*M* = 5.82, *SD* = 1.12) compared to South Asians (*M* = 5.34, *SD* = 1.44), *t*(149) = 5.04, *p* < .001, *d* = 0.41, 95% CI [0.29, 0.67]. Following a similar pattern to prototypicality ratings, White targets (*M* = 2.85, *SD* = 1.75) were less believed compared to East Asian, *t*(149) = 17.53, *p* < .001, *d* = 1.43, 95% CI [2.64, 3.30], and South Asian targets, *t*(149) = 15.25, *p* < .001, *d* = 1.24, 95% CI [2.17, 2.81], respectively.

Also replicating Study 2, when told the target was planning on reporting the crime on the SAAPI website, participants deemed it to be more appropriate for East Asian targets (*M* = 6.35, *SD* = 1.03) to do so compared to South Asian targets (*M* = 5.29, *SD* = 1.60), *t*(149) = 7.99, *p* < .001, *d* = 0.65, 95% CI [0.79, 1.31]. It was perceived to be less appropriate for White targets (*M* = 2.72, *SD* = 1.83) to use the reporting system compared to East Asians, *t*(149) = 20.30, *p* < .001, *d* = 1.66, 95% CI [3.27, 3.98], and South Asians, *t*(149) = 14.87, *p* < .001, *d* = 1.21, 95% CI [2.23, 2.91], respectively.

Contrary to Study 2, but in line with our original predictions, results of Study 3 indicated that participants believed East Asian targets (*M* = 5.90, *SD* = 1.02) would be significantly more traumatized if they were the victim of a hate crime compared to South Asian targets (*M* = 5.56, *SD* = 1.30), *t*(149) = 3.72, *p* < .001, *d* = 0.30, 95% CI [0.16, 0.53], and White targets (*M* = 4.60, *SD* = 1.88), *t*(149) = 8.57, *p* < .001, *d* = 0.69, 95% CI [1.00, 1.59], respectively.

**Figure 2.**
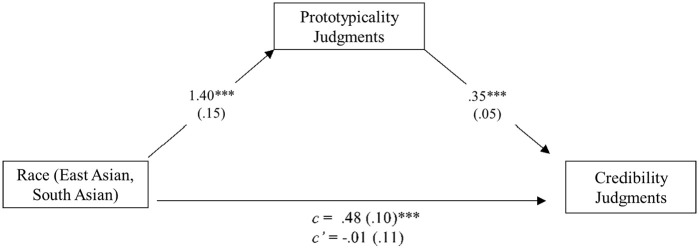
Model showing the effect of race on credibility judgments through perceived prototypicality: Study 3. *Note*. Indirect effect: *B* = 0.49, *SE* = 0.12, 95% CI [0.26, 0.75]. ***p < .001.

**Figure 3.**
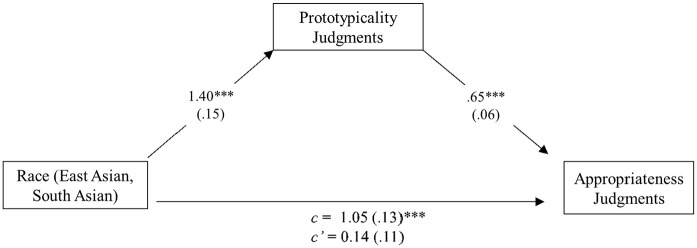
Model showing the effect of race on appropriateness judgments through perceived prototypicality: Study 3. *Note*. Indirect effect: *B* = 0.91, *SE* = 0.16, 95% CI [0.61, 1.23]. ***p < .001.

**Figure 4.**
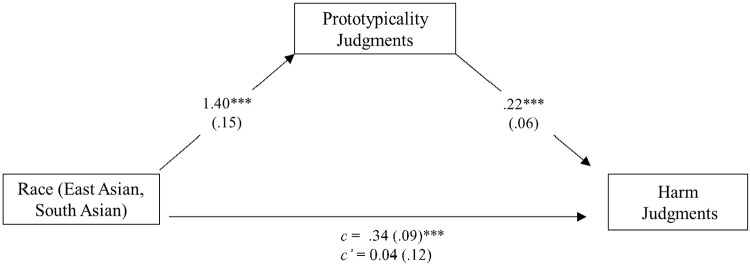
Model showing the effect of race on harm judgments through perceived prototypicality: Study 3. *Note*. Indirect effect: *B* = 0.30, *SE* = 0.12, 95% CI [0.08, 0.55]. ***p < .001.

The third goal of Study 3 was to test whether prototypicality judgments indirectly influenced the relationship between target race and perceptions of credibility, appropriateness, and harm. To address this hypothesis, we tested whether perceived prototypicality of the Stop Asian Hate movement mediated the relationship between target race and judged credibility (see Figure 2), appropriateness (see Figure 3), and harm (see Figure 4). Because our focus was on the comparisons between East Asian and South Asian targets, White targets were not included in the following mediation models. For each analysis, we used 10,000 percentile bootstrapped samples to test for a significant indirect effect. Results supported perceived prototypicality as mediating credibility, *B* = 0.49, *SE* = 0.12, 95% CI [0.26, 0.75]; appropriateness, *B* = 0.91, *SE* = 0.16, 95% CI [0.61, 1.23]; and harm, *B* = 0.30, *SE* = 0.12, 95% CI [0.08, 0.55]. Prototypicality was positively correlated with credibility, *r*(148) = .52, *p* < .001; harm, *r*(148) = .53, *p* < .001; and appropriateness, *r*(148) = .57, *p* < .001.

### Discussion

Replicating the previous studies, participants in Study 3 believed that East Asian targets were more represented by the Stop Asian Hate movement than South Asian targets. Further, when depicted as being the victim of a hate crime, East Asian targets were perceived to be more credible, more traumatized, and their reporting of the crime on the SAAPI website was deemed more appropriate, compared to South Asian targets who also claimed to be the victim of a hate crime. Of primary interest was whether beliefs about representativeness by the Stop Asian Hate movement drive this racial bias in credibility, harm, and appropriateness ratings. Results supported these hypotheses. Although these results are interesting and aligned with our predictions, we urge caution in making strong inferences from them. Despite existing literature supporting the potential mediator being theoretically motivated by existing effects (Goh et al., 2022), mediation cannot provide definitive evidence of causal relationships (e.g., Fiedler et al., 2018; Pek & Hoyle, 2016), plausible alternative mediators remain untested, and most effects are likely multiply determined. More research is needed to better understand the nuanced relationship between judgments of prototypicality, endured harm, credibility, appropriateness, and race, and to provide a more robust understanding of the mechanism(s) underlying the observed racial biases. Nevertheless, the current results suggest that perceptions of prototypicality may have an important role.

## General Discussion

Despite the heterogeneity among Asian subgroups, they are often monolithically categorized under the single racial label of Asian. This overgeneralization not only reduces the diversity of Asian subgroups, but also demonstrates a singular understanding of the broad racial group among some Americans. Research indicates that East Asians are perceived to be the most prototypically Asian group in the US (Goh & McCue, 2021). Substantial research has focused on the negative consequences of narrow prototypes (Phelan & Rudman, 2010; Purdie-Vaughns & Eibach, 2008; Schug et al., 2017; Sesko & Biernat, 2010; Vogel et al., 2021), however, limited research examines how these consequences apply to broad racial movements. The current work sought to fill this gap in the literature by examining whether people have narrow beliefs about which Asian subgroups are represented by the Stop Asian Hate movement and potential implications of such a prototypicality bias for several victim reporting metrics.

Study 1 provided initial evidence conceptually supporting previous research indicating an East Asian prototypicality bias among U.S. citizens (Goh & McCue, 2021). Compared to South Asian targets, participants perceived East Asian targets as more represented by the Stop Asian Hate movement. Study 2 tested potential implications of this bias by examining perceptions of credibility and experienced psychological harm following victimization (Goh et al., 2022). Results of Study 2 partially supported our hypotheses. When depicted as claiming to be the victim of a hate crime, East Asians’ claims were viewed as more credible, and it was deemed more appropriate for them to report their victimization on the SAAPI website, compared to South Asians’ claims. Surprisingly, no significant differences in perceptions of how traumatized the target would be emerged between South Asian and East Asian targets. Study 3 aimed to empirically test the mechanistic role of perceptions of representation by the Stop Asian Hate movement in perceptions of credibility, harm, and appropriateness of reporting on the SAAPI website following claims of hate crime victimization. Results of Study 3 replicated the previous studies, such that participants believed East Asian targets were more represented by the Stop Asian Hate movement than South Asian targets, and were perceived to be more credible, more traumatized (unlike Study 2), and their reporting of the crime on the SAAPI website was deemed more appropriate, compared to South Asian targets who also claimed to be the victim of a hate crime. Finally, Study 3 further demonstrated the role of perceptions of representativeness by the Stop Asian Hate movement in all three victim reporting metrics. Given the inconsistency in results on the traumatization item across Studies 2 and 3, more work is needed to provide a more complete understanding of this effect. Together, these studies consistently indicate an East Asian prototypicality bias in the US and demonstrate how this bias can negatively influence victims’ reporting experience.

### Implications

The current work extends foundational research on group prototypicality into a novel domain: social justice movements. Past work reliably demonstrates that having narrow prototypes can be problematic. Much of this work focuses on how women who do not represent the woman prototype due to their age, race, or sexuality are punished in myriad ways (e.g., Glick & Fiske, 2001; Goh et al., 2022; Kaiser et al., 2022; Purdie-Vaughns & Eibach, 2008). The current work contributes to research on racial prototypicality, with a particular focus on Asian racial identity. Although past work demonstrates that people have limited prototypes for who is considered Asian (J. Lee & Ramakrishnan, 2020), and that these effects are culturally bound (Goh & McCue, 2021), the current work links this literature to work on social justice movements. Specifically, these findings provide evidence for the problematic transference of narrow Asian prototypes in the US into beliefs about who is represented by the Stop Asian Hate movement. Limited perceptions of whom movements represent directly implicate who can credibly claim representation by the movement. Moreover, these perceptions may have implications for who is provided resources (e.g., funding, legal counsel), and who reaches out to receive support in the first place. Outgroup members, such as allies working with the organizations receiving the reports, or people in positions of power (e.g., legal professionals), may also evaluate these reports. Indeed, although ingroup members often act as gatekeepers for group membership, outgroup members, especially those in socially dominant positions, have had a long history of socially consequential judgments of others based on group membership vis-à-vis perceived prototypicality (e.g., Blair et al., 2002; Eberhardt et al., 2006).Thus, understanding intergroup evaluations is critical to ensure that low-prototypical Asian Americans not be inappropriately denied access to dedicated support resources. Further, although previous research suggests that ingroup members may actually be more sensitive to skin tone variations when compared to White perceivers (Strom et al., 2012), replications of the current project from an ingroup perspective are still needed.

Indeed, these findings have practical implications for the effectiveness and inclusiveness of broad social movements. Many racial social justice movements in the US, and globally, use broad racial language (e.g., Stop Asian Hate, Black Lives Matter). The use of broad language may be an intentional mechanism to be inclusive. Yet, when prototypes are narrow, seemingly inclusive language may ironically be exclusionary. These results demonstrate that narrow prototypes of the Asian racial category are problematic for South Asian people. Not fitting the prototype, our participants saw them as less represented by the Stop Asian Hate movement, and consequently found them less credible victims and thought they should not avail themselves of the SAAPI reporting resources compared to East Asian people. No simple solution is evident. Indeed, the Stop Asian Hate movement and specific organizations such as SAAPI position themselves as inclusive, using inclusive language and imagery. SAAPI allows participants to report a hate crime in one of 16 different languages. Yet as long as public perception of the category Asian largely refers to East Asian, others may feel excluded. Indeed, since the COVID-19 pandemic, Asian Americans report hate crimes at lower rates than other racial and ethnic groups in the US (Lantz & Wenger, 2022). Although a multifaceted issue, feeling as though reports may not be taken seriously or believed, contributes to these lower reporting rates.

### Limitations and Future Directions

Limitations in the current work can serve as potential avenues for future research. In the current work, we focused on how laypeople judge other people’s fit with the Stop Asian Hate movement. This tight focus afforded good experimental control around our specific research questions. However, it begs for at least two further questions. First, how does people’s racial and ethnic identification relate to self-perceptions of social justice movement representation? For example, do East Asian people feel more represented by Stop Asian Hate than South Asian people? And, does any difference in feeling represented predict reporting behaviours or trust in the movement itself? To the best of our knowledge, the limited work examining reporting (e.g., Lantz & Wenger, 2022) relies on the broad Asian label, suggesting an area for more nuanced inquiry. Second, we relied on a convenience sample that likely does not represent people who actually have power to act on hate crime reports. Understanding how legal professionals and police officers respond to victimization reports from people who vary in prototypicality is important to better understand how findings such as these might inform actual legal practices.

We specifically focused on East and South Asian targets within the context of the Stop Asian Hate movement. Extending this work to other people who identify as represented by this movement is important. Further, many other race-based social justice movements use broad racial language (e.g., Black Lives Matter) that might be influenced by prototypicality judgments. Future research should explore whether and how the prototype effects demonstrated in the current study translate to other movements beyond race. Expectations around gender, sexuality, or even environmentalism may have implications for movements such as #MeToo, Pride, or the March for Science. Importantly, as previous research has demonstrated gender-based biases in prototypicality judgements within the Asian population (Schug et al., 2015), it is possible these expectations may also influence patterns of victim reporting believability and appropriateness.

Finally, the current project focused solely on perceptions of U.S. participants. Race-based prototypes are culturally bound. For instance, recent research finds that South Asian is more prototypical than East Asian in the UK (Goh & McCue, 2021). It may be the case that culture moderates these findings. However, hate crimes against East Asian people have particularly skyrocketed following the COVID-19 pandemic, with media connections between the virus and East Asian countries fueling these attacks (Han et al., 2023; Tavernise & Oppel, 2020). Thus, it may be the case that the pandemic zeitgeist is as impactful, if not more, as prototypes in countries where East Asian is not the default. Regardless, future research should explore similar prototypicality effects on social justice movements in cultures around the globe.

## Notes

1. The use of the term “Asian American” in this context is due to the specific language on the SAAPI website. However, it is critical to note that the term “Asian American” is a political term, which, from its inception, intended to promote pan-ethnicity and coalition building. Although contemporary American understandings of the term often narrowly focus on individuals with East Asian heritage, it is essential to consider the broader context of its origins. In the current paper, the use of the term “Asian” is intended to encompass both individuals born in Asia as well as individuals born in America with Asian heritage.

2. Note that the 95% confidence intervals reported throughout the current manuscript are structured around the mean difference of the sample estimate.
